# Assessing the prevalence and association between physical, emotional, and sexual of intimate partner violence against women in Nigeria

**DOI:** 10.1186/s12978-022-01431-9

**Published:** 2022-06-23

**Authors:** Lijalem Melie Tesfaw, Essey Kebede Muluneh

**Affiliations:** 1 Department of Statistics, Bahir Dar University, Bahir Dar, Ethiopia; 2 School of Public Health, Bahir Dar University, Bahir Dar, Ethiopia

**Keywords:** Violence against women, Log-linear model, Nigeria

## Abstract

**Background:**

Several studies were carried out on prevalence and associated factors of physical, emotional, and sexual violence against women. However, little attention was given to a comprehensive study that assesses the association between physical, emotional, and sexual violence against women. Thus, this study aimed to assess the association between physical, emotional, and sexual violence against women and their prevalence.

**Methods:**

A retrospective cross-sectional design was implemented based on the 2018 Nigeria Demographic and Health Survey involving 8061 married women aged 15–49. A log-linear statistical model for the three-way table was used to assess the association between emotional, physical, and sexual violence. SAS statistical software was used for data management and parameter estimation.

**Results:**

Among a total of 8061 women considered in the study 3022 (37.49%), 4216 (52.3%) and 1186 (14.71%) women have experienced physical, emotional, and sexual violence, respectively. The estimated odds of the interaction between emotional and physical violence (e^**1.9281**^ = 6.876); physical and sexual violence (e^**−2.0529**^ = 0.128) were significantly differ from 1.0 with p-values < 0:0001 and 0.0201, respectively.

**Conclusion:**

Over 33 percent of women experienced at least one incident of physical, emotional, or sexual violence in their lifetime. Physical violence against women has a significant association with emotional and sexual violence. However, it does not imply physical violence causes the other violence since cross-sectional data used for the analysis and other factors were not taken into consideration. The lack of a three-way association between emotional, physical, and sexual violence was also perceived. Therefore, as the prevalence of intimate partner violence against women s high, Nigeria as a country needs to strive to reduce it with the collaboration of other nations in the world to achieve Sustainable Development Goal (SDG). Design and apply guidelines to aware of the community about intimate partner violence against women and besides, take appropriate sentencing on those who commit the violence are the better approaches to prevent violence. Traditional habits that might be the cause of violence should be avoided to reduce or prevent the burden of women due to violence.

## Plain language summary

Even though studies were carried out on physical, emotional, and sexual violence against women, and prevalence and associated factors, little attention was given to a comprehensive study that assesses the association between physical, emotional, and sexual violence against women. As a result, this study aimed to assess the association between physical, emotional, and sexual acts of violence against women in Nigeria. The data was obtained from the 2018 Nigeria Demographic and Health Survey (NDHS). A total of 8061 ever married/cohabited women aged 15–49 years who were residents of the study community for at least 6 months were considered. The log-linear statistical model was used to assess the association between physical, emotional, and sexual violence against women.

Of the 8061 women considered in this study, 516(6.40%) have ever been physically forced into unwanted sex by a husband/partner. About 1346 (16.70%) and 1467(18.20%) women have ever been slapped and humiliated by their husbands/partners, respectively. Whereas 3022 (37.49%), 4216 (52.3%), and 1186 (14.71%) women have experienced physical, emotional, and sexual violence, respectively. Four hundred sixty-six women were victims of physical, emotional, and sexual violence. Besides, 40, 755, and 144 women have experienced both physical and sexual violence; emotional and physical violence; and emotional and sexual violence respectively. Physical violence against women has a significant association with emotional and sexual violence. However, it does not imply physical violence causes the other violence since cross-sectional data used for the analysis and other factors were not taken into consideration.

## Introduction

The UN Declaration on the Elimination of Violence against Women (DEVW), defines the term “Violence against women” means any act of gender-based violence that results in, or is likely to result in, physical, sexual, or psychological harm or suffering to women, including threats of such acts, coercion or arbitrary deprivation of liberty, whether occurring in public or private life [1]. Usually, acts of violence against women are measured in three dimensions physical, emotional, and sexual violence [2]. Globally, the most common (85%) form of violence against women is executed by a husband or other close partners. According to the World Health Organization (WHO), domestic violence, mainly intimate partner violence, has a significant component of gender-based violence [1, 2]. Intimate personal violence refers to any behavior within an intimate relationship that causes physical, psychological, or sexual harm to those in the relationship [3]. Thirty percent of women aged 15 and above have experienced physical and sexual violence by their partners in the world. Whereas, seven percent of women have encountered sexual violence by non-partners 27.5 percent of women were with physical and emotional violence [4].

In Europe, 20% of women have a victim of physical or sexual intimate partner violence [5]. Whereas both physical and emotional violence was experienced by 22.5% of the women., About 9.6% of women in the study encountered sexual violence, indicating that physical and emotional violence has taken the lead followed by physical and emotional violence was encountered by 22.5% of women, while sexual violence was 9.6% of women. This indicates that physical and emotional violence is predominant. In parallel to the study [6], compared to physical or sexual violence, physiological violence is the most prevalent type of violence against women.

The proportion of ever-partnered women who had ever suffered physical violence by a male intimate partner ranged from 13% in Japan city to 61% in Peru province, with most sites falling between 23 and 49% while the range of lifetime prevalence of physical or sexual violence, or both, by an intimate partner was 15–71%, with estimates in most sites ranging from 30 to 60% and women in Japan city being the least likely to have ever experienced physical or sexual violence, or both, by an intimate partner [7].

Intimate partner violence against women has a significant impact on the empowerment of women. Compared to women who have not experienced intimate partner violence, women who have experienced intimate partner violence are feeling discouraged and hopeless. The most common foundation of physical, psychological, and emotional morbidity of women is because of unavoidable violence against them [8]. There are numerous forms of violence against women [9]. The three acts of violence against such as physical, emotional, and sexual violence have been associated with health concerns including sexual, physical, reproductive health, behavioral and psychological problems [10]. Commonly emotional violence against women is an intrinsic aspect of both physical and sexual violence against women. A study conducted in Nicaragua [11] revealed a considerable coexistence between physical, emotional, and sexual violence. The findings of a study in Iraq was also reported that a high prevalence of intimate partner violence, in particular, emotional violence is the prevalent violence. More than half of women were victims of emotional, physical, and or sexual violence at least once by their husbands, and 45.3% gave a history of persistent abuse [12]. Though gender-based violence is a global health problem, it is higher in developing countries like Africa [9]. As a result of traditional, religious, and others factors in the communities, the highest proportion of intimate partner violence against women is existed in Africa [12]. Women living in Eastern and Western African regions experience the highest levels of gender-based violence [8]. A study involving the 2016 Ethiopian Demographic and Health Survey showed that still a high prevalence of domestic violence in the form of physical, emotional, or sexual violence against women from an intimate partner.

Community-based studies on pregnant women reported that the prevalence of domestic violence ranged from 32.2 to 45.5 percent. Besides, 25.4 percent overall prevalence of domestic violence such as physical, emotional, and sexual violence was 8.1, 24.5, and 2.4% respectively in Gondar town, Ethiopia [13]. Almost one-third of Ethiopian women aged 15–49 experienced at least one type of violence in their lifetime and one-third of women globally [14].

A study in Northern Nigeria revealed that almost sixteen percent of female university students experienced one or more gender-based violence committed. In particular, students experienced physical (22.8%), sexual (22.2%), and emotional violence (50.8%). In this study, it is also reported that almost thirty percent of students were exposed to pressure by their teachers for sex, and eighteen percent were usually harassed by their classmates [15]. A study in [9] found that intimate personal violence in developing countries is high. However, often it is difficult to identify women at risk because of a culture of silence within the countries. Over two-fifths of women reported experiencing any intimate parent violence against women in Angola. Of these 32.3, 27.3 and 7.4% are physical, emotional, and sexual violence respectively which may occur between the same or different sex [16]. Though the Universal Declaration of Human rights declares that all people need to be recognized irrespective of gender, women have continued to suffer from intimate partner violence. The Declaration tried to investigate in different periods to determine the recent trends of prevalence of intimate partner violence against women [13, 14]. However, due to cultural, and other restrictions, the government leads to giving less attention. African countries have taken the highest proportion including the largest populist country in the continent, Nigeria. The Nigerian community is a masculine community that considers husbands’ ability over women in the household which aggravates the occurrence of violence against women [17].

To date, numerous studies have been done on the causes of physical, emotional, and sexual violence against women such as socioeconomic and demographic determinants [4, 5, 15, 18]. However, to the best of the authors’ knowledge, no published literature assessed the association between physical, emotional, and sexual violence against women in the world. Moreover, there is a lack of study findings determining the overall prevalence of intimate partner violence against women in terms of emotional, sexual, and physical violence [2, 3]. Little attention was given to a comprehensive study that assesses the association between physical, emotional, and sexual violence against women in Nigeria as well. Therefore, this study aimed to assess the association between physical, emotional, and sexual acts of violence against women in Nigeria. Finding out the association between physical, emotional, and sexual violence against women has numerous rationale to achieve the Sustainable Development Goal (SDG) and to produce mothers who enable to care for children without being a victim by their intimates. This leads to having a good generation that enables solving problems in the family based on evidence and knowledge rather than committing violence. Thus, the findings of this study will benefit policymakers at governmental and private levels directly in Nigeria. Moreover, it will have an input for countries outside of Nigeria to conduct similar research papers and incorporate findings on temporary and permanent strategies. Therefore, the study findings on intimate violence against women are a crucial input to develop evidence-based national and regional intimate violence prevention policies and strategies for governmental and non-governmental organizations. Besides, it can act as an input for future researchers regarding violence against women and build up a concrete remedial action that enables to reduce the prevalence of victim women due to their parents/intimate friends.

## Methods

### Data

A cross-sectional, population-based household survey involving data from the 2018 Nigerian Demographic and Health Survey (NDHS) was used. The 2018 NDHS data collection took place between 14 August and 29 December 2018, via a stratified two-stage cluster sample design using a sampling frame containing the list of enumeration areas prepared for the 2006 Population Census of the Federal Republic of Nigeria [19]. In the first stage, enumeration areas were selected proportional to the enumeration area size. In the second stage, a fixed number of households were selected in every cluster through equal probability systematic sampling. The 2018 NDHS data used was designed to provide up-to-date estimates of basic demographic and health indicators. It is intended to assist policymakers and program managers in evaluating and designing programs and strategies for improving the health of the country’s population [1, 19].

In this study 8061 ever married/cohabited women aged 15–49 years who were residents of the study community for at least 6 months were considered. A module of questions designed to obtain information on the extent to which women in Nigeria experience intimate partner violence was used. Only one eligible ever married/cohabited woman age 15–49, who was the resident of the study community for at least 6 months, per household was randomly selected for the module, and the module was not implemented if privacy could not be obtained [1, 20]. The violence committed by the husband or close partner was measured by asking all ever-married women if their husband/partner ever did the following to them [1, 13, 14]:

*Physical violence*: Push you, shake you, or throw something at you; slap you; twist your arm or pull your hair; punch you with his fist or with something that could hurt you; kick you, drag you, or beat you up; try to choke you or burn you on purpose; or threaten or attack you with a knife, gun, or any other weapon.

*Sexual violence*: Physically force you to have sexual intercourse with him even when you did not want to, physically force you to perform any other sexual acts you did not want to, or force you with threats or in any other way to perform sexual acts you did not want to.

*Emotional violence*: Say or do something to humiliate you in front of others, threaten to hurt or harm you or someone close to you, or insult you or make you feel bad about yourself.

In this study, he references papers that show statistical results related to victimization from 2010 till 2022 were considered.

### Inclusion/exclusion criteria

The inclusion criteria were women aged 15–49 years, who have an intimate partner and completed relevant forms about the personal information and clinical signs. Hence, women who had not completed all relevant information or aged 15–49 years or women who don’t have intimate partners were excluded.

### Ethics approval

NDHS Program granted permission to download and use the data for this study after being registered and submitting a request with briefly stated objectives of the study. The Institution Review Board approved procedures for DHS public-use data sets that do not in any way allow respondents, households, or sample communities to be identified. There are no names of individuals or household addresses in the data files. The detail of the ethical issues has been published in the 2018 NDHS final report, which can be accessed at:http://www.dhsprogram.com/publications.

### Limitation of the study

This study is conducted based on cross-sectional data and hence not assessed the prevalence of intimate partner violence against women. Besides, other socioeconomic, demographic, biological, and behavioral characteristics were not considered. Thus, we authors would like to recommend if future researchers considered these characteristics as they might affect violence against women.

## Statistical methods

### Log-linear model

Log-linear model is a family of generalized linear models used to model cell counts in contingency tables. It is used to specify the association patterns among a set of categorical response variables i.e., how the size of a cell count depends on the levels of the categorical variables. In the log-linear model, the null hypothesis states that there is no association between the two variables in the contingency table and the alternative hypothesis stipulates the existence of an association between the two variables. To use a log-linear model, there should be at least two response variables in the contingency table [17].

In the case of the log-linear model for three-way contingency tables, log-linear models can represent various independence and association patterns [21]. The number of all possible log-linear models for k categorical variables is 2^* k*^ + 1 [17, 22]. Consequently, in this study, we will have 2^3^ + 1 = 9 possible log-linear models to be fitted (see Table 1). In the table, all possible log-linear models starting from the simplest (pair of variables are independent, both conditionally and marginally) to advanced complex (all possible interactions, that is, the interaction between emotional and physical violence; emotional and sexual violence; physical and sexual violence; emotional, physical and sexual violence) were presented. Even though log-linear models are appropriate to test hypotheses about complex interactions, it is not easy to interpret the parameter estimates. Parameter estimates are log odds ratios for associations [22].

**Table 1:** Possible log-linear models of the three-way table of Emotional (E), Physical (P), and Sexual (S) violence

Model expression	Description
$log(\mu_{ijk})=\lambda + \lambda ^{E}_{i}+ \lambda^{P}_{j}+\lambda^{S}_{k}$	Mutual independence model (pair of variables are independent, both conditionally and marginally)
$log(\mu_{ijk})=\lambda + \lambda^{E}_{i} + \lambda^{P}_{j} +\lambda^{S}_{k}+ \lambda^{EP}_{ij}$	Sexual violence is partially independent of Emotional and Physical violence. This model contains the interaction between Emotional and Physical violence
$log(\mu_{ijk})=\lambda + \lambda^{E}_{i} + \lambda^{P}_{j} +\lambda^{S}_{k}+ \lambda^{ES}_{ik}$	Physical violence is partially independent of Emotional and Sexual violence. This model contains the interaction between Emotional and Sexual violence
$log(\mu_{ijk})=\lambda + \lambda^{E}_{i} + \lambda^{P}_{j} +\lambda^{S}_{k}+ \lambda^{PS}_{jk}$	Emotional violence is partially independent of Physical and Sexual. This model contains the interaction between Physical and Sexual violence
$log(\mu_{ijk})=\lambda + \lambda^{E}_{i} + \lambda^{P}_{j} +\lambda^{S}_{k}+ \lambda^{EP}_{ij}+\lambda^{ES}_{ik}$	Physical and Sexual violence are conditionally independent of Emotional. This model contains the interaction terms between Emotional and physical violence; Emotional and Sexual violence
$log(\mu_{ijk})=\lambda + \lambda^{E}_{i} + \lambda^{P}_{j} +\lambda^{S}_{k}+ \lambda^{EP}_{ij}+\lambda^{PS}_{jk}$	Emotional and Sexual violence are conditionally independent of Physical violence. This model contains the interaction terms between Emotional and Physical violence; Physical and Sexual violence
$log(\mu_{ijk})=\lambda + \lambda^{E}_{i} + \lambda^{P}_{j} +\lambda^{S}_{k}+ \lambda^{ES}_{ik}+\lambda^{PS}_{jk}$	Emotional and Physical violence are conditionally independent of Sexual violence. This model contains the interaction terms between Emotional and Sexual violence; Physical and Sexual violence
$log(\mu_{ijk})=\lambda + \lambda^{E}_{i} + \lambda^{P}_{j} +\lambda^{S}_{k}+ \lambda^{EP}_{ij}+\lambda^{ES}_{ik} + \lambda^{PS}_{jk}$	Homogenous associations (every violence of the three interacts with each other, but there is no interaction between all three violence)
$log(\mu_{ijk})=\lambda + \lambda^{E}_{i} + \lambda^{P}_{j} +\lambda^{S}_{k}+ \lambda^{EP}_{ij}+\lambda^{ES}_{ik} + \lambda^{PS}_{jk} + \lambda^{EPS}_{ijk}$	All possible interaction between violence

log(*µ*_*ijk*_) = log of expected cell counts; E = Emotional; P = Physical; S = Sexual; *λ*^*E*^_*i*_*,λ*_*j*_^*P*^*,λ*^*S*^_*k*_ = the effect of Emotional, Physical and Sexual violence respectively. Similar for the interaction term. For instance, *λ*^*EP*^_*ij*_ represent the interaction effect among Emotional and Physical violence

The model fitted by equating the log of expected cell counts *µ*_*ijk*_. The expected cell count is the combination of the status (yes, no) of the three variables of women on that cell. The subscript i, j, and k represent emotional, physical, and sexual violence, respectively.

### The goodness of fit test

The goodness of fit test is used to assess the distance between the observed distribution and the distribution that the proposed model, see Table 4. Statistical tests commonly used to test the goodness a fit of a log-linear model are Chi-squared statistic (*χ*^2^) and likelihood ratio statistic (*G*^2^) [17].

PROC GENMOD procedure in SAS 9.4 was used to fit the model and decisions were made using a 0.05 level of significance.

## Results

The violence against women considered in this study (sexual, physical, and emotional violence) was measured from a combination of different latent variables, see Table 2. For instance, to measure whether the women experienced emotional violence, questions such as humiliated, threatened with harm, and insulted or made to feel bad by husband/partner were included. If the woman has experienced at least one of these forms of violence, then the woman was considered a victim of emotional violence. The same procedures were used for physical and sexual violence.

**Table 2:** Prevalence of different forms of sexual, physical, and emotional violence against women in Nigeria

Violence against women	Frequency (%)
Sexual violence	
Ever been physically forced into unwanted sex by your husband/partner	516 (6.40)
Ever been forced into other unwanted sexual acts by your husband/partner	210 (2.60)
Ever had arm-twisted or hair pulled by your husband/partner	210 (2.60)
Ever been physically forced to perform sexual acts respondent didn’t want to	250 (3.10)
Physical violence	
Ever been pushed, shook, or had something thrown by your husband/partner	556 (6.90)
Ever been slapped by your husband/partner	1346 (16.70)
Ever been punched with the fist or hit by something harmful by a husband/partner	331 (4.10)
Ever been kicked or dragged by husband/partner	677 (8.40)
Ever been strangled or burnt by a husband/partner	64 (0.80)
Ever been threatened with a knife/gun or another weapon by husband/partner	48 (0.60)
Emotional violence	
Ever been humiliated by your husband/partner	1467 (18.20)
Ever been threatened with harm by your husband/partner	484 (6.00)
Ever been insulted or made to feel bad by your husband/partner	2265 (0.28)

Of the 8061 women considered in this study, 516(6.40%) have ever been physically forced into unwanted sex by a husband/partner. About 1346 (16.70%) and 1467(18.20%) women have ever been slapped and humiliated by their husbands/partners, respectively. Among a total of 8061 women considered in the study, 3022 (37.49%), 4216 (52.3%), and 1186 (14.71%) women have experienced physical, emotional, and sexual violence, respectively. Here, caution needs to be taken for the interpretation of the total population as women might have experienced more than one of the three acts of violence.

Four hundred sixty-six women were victims of physical, emotional, and sexual violence. On the other hand, 40, 755, and 144 women have experienced both physical and sexual violence; emotional and physical violence; and emotional and sexual violence respectively, see Table 3. For better clarification, the frequency distribution of women among the three acts of violence was also presented in Table 4.

**Table 3:** Cross-tabulation of emotional, physical and sexual violence

Emotional			Sexual		Total
			No	Yes	
No	Physical	No	5049	85	5134
		Yes	305	40	345
Yes	Physical	No	1217	144	1361
		Yes	755	466	1221
Total			7326	735	8061

**Table 4:** Non-overlapping categories of emotional, physical, and sexual violence against women in Nigeria

Category	Frequency (%)
None	5049 (62.63)
Emotional only	1217 (15.10)
Physical only	305 (3.78)
Sexual only	85 (1.05)
Emotional and physical	755 (9.37)
Emotional and sexual	144 (1.79)
Physical and sexual	40 (0.50)
Emotional, physical and sexual	466 (5.78)

In Table 4, a woman involved in this study was categorized into none (women experienced neither of the three acts of violence); emotional only; physical only; sexual only; emotional and physical; emotional and sexual; physical and sexual; emotional, physical, and sexual violence. This is important to determine the women experienced more than one violence throughout their life. For instance, the number of women who experienced physical and emotional violence was 755 (9.37%). Moreover, 5.78 percent of the women were experienced emotional, physical, and sexual violence. Table 4 revealed that more than one in three (37.37% = 100- 62.63%) of the women included in the study encountered emotional, sexual, and/or physical violence (see Table 4).

The proportion of women who have experienced physical and sexual violence (0.50%) was the lowest compared to women in the other violence category. Whereas women experienced emotional violence (15.10%) was the highest followed by women who experienced physical and emotional violence (9.37%).

To fit the model and estimate the parameters first goodness of fit test of all possible log-linear models needs to be checked. The goodness of fit test for the fitted log-linear models of cross-tabulation of emotional, physical, and sexual violence was depicted using Pearson Chi-square (*χ*^2^) and likelihood ratio (LR) statistic in Table 5. The null hypothesis states that the model is a good fit for the data, in a sense that the observed and the fitted cell counts are the same. And the reverse claims are true for the alternative hypothesis.

**Table 5:** Goodness-of-fits tests for log-linear models of emotional (E), physical (f), and sexual (c) violence against women in Nigeria

Model	Log linear model	LR	Chi	Df	P-value	
					LR	Chi
1	(E,P,S)	5361.41	5314.62	4	< 0.0001	< 0.0001
2	(EP,S)	5306.49	1594.32	3	< 0.0001	< 0.0001
3	(P,ES)	5214.36	2370.20	3	< 0.0001	< 0.0001
4	(E,PS)	4345.85	2144.16	3	< 0.0001	< 0.0001
5	(EP,ES)	2309.44	414.65	2	< 0.0001	< 0.0001
6	(EP, PS)	1340.93	338.76	2	< 0.0001	< 0.0001
7	(PS,ES)	1598.80	1332.13	2	< 0.0001	< 0.0001
8	(EP, ES,PS)	40.66	3.14	1	< 0.0001	< 0.0001
9	(EPS)	0.000	0.000	0	1.000	1.000

*LR* likelihood ratio, Chi = *χ*^2^(Pearson Chi-square), *Df* degree of freedom, *E* emotional, *P* physical, *S* sexual

The higher values of Pearson Chi-square (*χ*^2^) and likelihood ratio (LR) corresponding with a p-value less than 0.05 significant level for the fit statistics indicates stronger evidence against the model fits the data well. However, a fitted model with a p-value greater than 0.05 supports the null hypothesis, indicating that the adapted model fits the data well. Table 5 depicts that the p-values of the fit statistics of Model 1 to Model 8 is much less than 0.05 level of significance indicating that the models do not fit the data well. However, the p-values for the fit statistics of Model 9 is much greater than the 0.05 level of significance (1.00) indicating that Model 9, the saturated model, fits the data well. Once the best fit model (saturated) of the data was identified, parameter estimates of the saturated model were fitted in Table 6.

**Table 6:** Parameter estimates of the saturated log-linear model consists of emotional, physical, and sexual violence against women in Nigeria

Parameter	Estimate	Standard error	Chi-Square	p-value
Intercept	4.4427	0.1085	167.66	< 0.0001
Emotional	0.5272	0.1368	14.85	0.0001
Physical	− 0.7538	0.1917	15.45	< 0.0001
Sexual	4.0843	0.1094	134.45	< 0.0001
Emotional × physical	1.9281	0.2141	81.08	< 0.0001
Physical × sexual	− 2.0529	0.2006	10.72	< 0.0201
Emotional × sexual	− 0.450	0.1405	2.73	0.0771
Emotional × physical × sexual	0.3011	0.1269	0.120	0.6721

In the fitted saturated model in Table 6, the null hypothesis of each coefficient of the interaction term is stated as there is no interaction among the two acts of violence. A p-value of the estimated coefficient of interaction term less than the significance level (0.05) indicates significant interaction between the two acts of violence. Hence, Table 6 shows a high interaction between emotional and physical (emotional × physical) violence; and physical and sexual (physical × sexual) violence as the p-value less than 0.05 (< 0*.*0001). The estimated odds between emotional and physical violence [exp(1.9281) = 6.876]; physical and sexual violence [exp(− 2.0529) = 0.128] significantly differed from 1.0 with p-values < 0*.*0001 and 0.0201 respectively which indicates that there is an association between the two acts of violence against women. In the last two rows, however, the saturated log-linear model shows a lack of association between emotional and sexual violence (p-value = 0.0771), and there was also a lack of three-way association between emotional, physical, and sexual violence since p-value much greater than 0.05 (p-value = 0.6721).

**Fig. 1:**
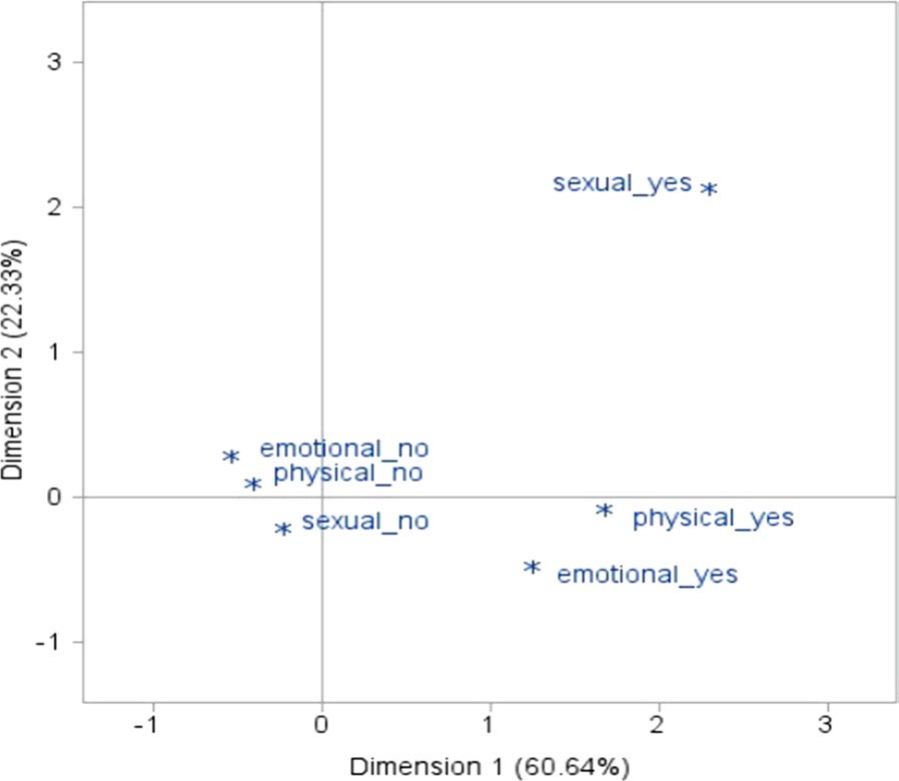
Plot of multiple correspondence analysis of emotional, physical, and sexual violence against women in Nigeria

The association between the three acts of violence against women can also be presented in multiple correspondence analysis plots beyond the model fitted in Table 6, see Fig. 1. The multiple correspondence analysis plots, in Fig. 1, suggest that there is a visible association between emotional and physical violence against women as indicated in the second and fourth quadrants. In the figure, the Asterix of physical violence closer to the horizontal boundary line indicates that physical violence also has an association with sexual violence in addition to emotional violence against women. This result supports the association between violence against women in Table 6 from the fitted saturated model, indicating that there is a significant association (p-value less than 0.05) between emotional and physical violence and physical and sexual violence against women.

## Discussion

This study has been done to demonstrate the prevalence of emotional, physical, and sexual violence against women and explore the association between them. The proportion of women who have experienced violence was presented using a cross-tabulation table. Emotional violence against women was found to be the most prevalent (52.3%) of all women aged 15–49 considered in the study, which is consistent with the study reported in Iraq as emotional violence is the most common type of violence [9, 12]. In this study, it was also found 37.49% and 14.71% of women had experienced physical and sexual violence respectively in their lifetime. The prevalence of violence in this study (37.37%) is greater than the prevalence reported in Gondar town, Ethiopia (25.4%) as well as all over Ethiopia (32.5%) [15, 23]. Therefore, the overall prevalence of violence against women aged 15–49 who experienced at least one form of intimate partner violence was found to be 37.37%. This prevalence is lower than the prevalence reported in Northern Nigeria female university students (58.8%) who have been experienced any form of gender-based violence [15] and the prevalence in comprehensive national analysis in Ethiopian women found that nearly half of all women experiencing lifetime violence against women [6] and Zimbabwe (61.3%) [13]. This reduction of violence might be an input to achieve the theme of sustainable development goals (SDG) which states that every country is expected to be intimate partner violence-free and eliminate all forms of violence against women in 2030 [14, 24]. As the prevalence of intimate partner violence against women in Nigeria is high (over 33%), Nigeria as a country needs to strive to reduce it with the collaboration of other nations in the world to achieve SDG. Design and applying guidelines to aware of the community about intimate partner violence against women would be more effective to reduce the occurrence of violence. If still, violence is taking place, taking appropriate sentencing on those who commit the violence to teach the community is necessary. Nevertheless, the inconsistency of prevalence might be because almost all the women joined at university are younger than women in this study aged 15–49. The wide inconsistencies in the prevalence of violence may reflect different definitions for violence in every society, the method of screening, religious beliefs, and cultural issues and it could be due to the differences in the time frame because of globalization/civilization or scope of gender-based assessment and differences in socio-cultural characteristics [15].

Almost one in ten (9.37%) women aged 15–49 exposed to emotional violence are a victim of physical violence. In this study, women experienced with more than one violence was perceived. In this regard, more than five percent of the women were victims of physical, emotional, and sexual violence. More than 37% of women included in the study were exposed to sexual, emotional, and/or physical violence. It is a high prevalence of violence against women as compared to global and regional estimates of violence against women [25]. This might be sometimes because of the culture, beliefs, and attitudes of the society that is living in the nation. In Nigeria, intimate personal violence is widely forgiven in many Nigerian societies where the belief that it is tolerable for the husband to discipline his wife is deeply embedded in the culture [10]. Thus, women have been entertained to accept and sometimes to encourage physical abuse. Due to this and other traditional habits, women are affected by physical, emotional, and sexual violence. As a result, it is highly suspected that a higher number of women might be experienced violence than the prevalence reported in this study (37%) except for underreporting and lack of investigation problems.

A study result found in Spanish Macrosurvey [5] reported that violence against women may be the cause of another health problem. Intimate partner violence harms women’s mental health, physical health, and daily activity. About 15.47% of women who had experienced any violence reported experience of all the three acts of violence which is higher (10%) compared to a result found in [18]. However, women who had experienced emotional and physical violence (25.07%) were lower than women who had experienced emotional and physical violence in [18]. This coexistence of different forms of violence leads to suspect if there is an association between them. To detect whether there is a significant association between violence, a statistical model which is called the log-linear model was implemented.

In the log-linear model, the association among the three acts of violence was represented using the interaction term. Log-linear models have the advantage of assessing the three-way interaction [17], beyond the very common analysis of pairwise association. As compared to the unsaturated log-linear model, the expected cell counts of emotional, physical, and sexual violence were well fitted (p-value greater than 0.05) by the saturated log-linear model. In the fitted model as well as multiple correspondence analysis plots (Fig. 1) of emotional, physical, and sexual violence it was observed that physical violence is significantly associated with both emotional and sexual violence. This is consistent with a study done by [10], which revealed that women who had experienced physical intimate personal violence were more likely to have experienced psychological and sexual violence when compared with their counterparts who had not experienced physical intimate personal violence. A study done by [9] reported that the coexistence of emotional violence with both physical and sexual violence is common in most reproductive-aged women. This concurrence of physical, sexual, and emotional violence indicates the association between violence. Thus, taking remedial action taken on one of the violence will help to overcome the other as well. Substantial overlap between physical, emotional, and sexual violence with 21% of ever-married women reporting all three kinds of violence indicated in Nicaragua [11], which was higher compared to women reporting three kinds of violence in this study (14.7%), in Nigeria. These research findings will be a good input for decision-makers and other stakeholders in Nigeria. The authors would like to recommend that further research be conducted not only in Nigeria but also in other African countries to have better evidence about violence against women in the continent.

## Conclusion

In conclusion, over 37 percent of women experienced at least one incident of physical, emotional, or sexual violence in their lifetime. Physical violence against women has a significant association with emotional and sexual violence. However, it does not imply physical violence causes the other violence since cross-sectional data used for the analysis and other factors were not taken into consideration. The study also concludes that emotional and sexual violence is not associated. Moreover, there is no three-way association between emotional, physical, and sexual violence. This implies that the three acts of violence have a multidimensional nature. Therefore, policy and decision-makers at a different level of leadership should consider the three acts of violence simultaneously to estimate the actual burden of violence against women as they are not redundant to each other. Finally, the author would like to recommend if a further study was conducted to assess the causal relationship between the three acts of violence by incorporating determinants of wife and husband such as educational status, age, religion, and other characteristics.

## Data Availability

The datasets for generated analyses during the study is available in Nigerian Demographic and Health Survey data if the unique request sent via their site NDHS 2018.

## Abbreviations

NDHS: Nigeria Demographic Health Survey
LR: Likelihood ratio
WHO: World Health Organization
UN: United Nation
DEVW: Declaration on the elimination of violence against women
SAS: Statistical analysis system
